# Effects of Drying Methods on Physicochemical and Immunomodulatory Properties of Polysaccharide-Protein Complexes from Litchi Pulp

**DOI:** 10.3390/molecules190812760

**Published:** 2014-08-20

**Authors:** Fei Huang, Yajuan Guo, Ruifen Zhang, Yang Yi, Yuanyuan Deng, Dongxiao Su, Mingwei Zhang

**Affiliations:** 1 Department of Food Science and Technology, Huazhong Agricultural University, Wuhan 430070, China; 2 Sericultural and Agri-food Research Institute, Guangdong Academy of Agricultural Sciences, Guangzhou 510610, China; 3 College of Food Science & Engineering, Wuhan Polytechnic University, Wuhan 430023, China

**Keywords:** litchi pulp, polysaccharides, drying method, immunomodulatory activity

## Abstract

Dried litchi pulp has been used in traditional remedies in China for many years to treat various diseases, and the therapeutic activity has been, at least partly, attributed to the presence of bioactive polysaccharides. Polysaccharide-protein complexes from vacuum freeze-(VF), vacuum microwave-(VM) and heat pump (HP) dried litchi pulp, which were coded as LP-VF, LP-VM and LP-HP, were comparatively studied on the physicochemical and immunomodulatory properties. LP-HP had a predominance of galactose, while glucose was the major sugar component in LP-VF and LP-VM. Compared with LP-VF and LP-VM, LP-HP contained more aspartate and glutamic in binding protein. LP-HP also exhibited a stronger stimulatory effect on splenocyte proliferation at 200 μg/mL and triggered higher NO, TNF-α and IL-6 secretion from RAW264.7 macrophages. Different drying methods caused the difference in physicochemical properties of polysaccharide-protein complexes from dried litchi pulp, which resulted in significantly different immunomodulatory activity. HP drying appears to be the best method for preparing litchi pulp to improve its immunomodulatory properties.

## 1. Introduction

Litchi (*Litchi chinensis* Sonn.), a fruit originating from China, is now planted widely in subtropical areas worldwide. The popularity of litchi is not only due to its attractive appearance and delicious flavor, but also its high nutritional value [1]. Besides some biological phytochemicals from litchi [2,3,4], the polysaccharides with immunostimulatory and antioxidant activities, has been more and more attractive [5,6,7]. 

Litchi is a seasonal fruit and has a short storage life due to its perishable nature [8], and drying is the most convenient method for storage. In addition to conventional hot air drying, several new drying techniques have been applied to treat litchi pulp, which include vacuum freeze (VF) drying, vacuum microwave (VM) drying and heat pump (HP) drying, *etc.* VF drying is a popular method for removing water from highly heat-sensitive materials without dramatically altering the physico-chemical properties of the sample. However, this technique is a time and energy consuming process and requires specific drying equipment [9]. VM drying has been investigated as a potential method for obtaining high quality dried food products, due to its higher drying rate and lower drying temperature on account of the application of microwave [10]. Compared to conventional hot air drying, VM drying could reduce the drying time by 70%–90% [11]. HP drying is also widely used in the food industry as it offers good product quality control and has a low energy consumption and environmental impact [12,13].

It is well known that different drying processes have different effects on the physico-chemical properties of materials, as well as the functional properties of their active ingredients because of the distinction of temperature, drying time and other conditions. These parameters have been reported to affect the structure and biological activity of the polysaccharides [14]. Freeze-dried *Inonotus obliquus* polysaccharides had a lower molecular weight and exhibited superior antioxidant activities compared to hot air- and vacuum-dried polysaccharides [15]. Fan *et al.* [16] reported that the VF drying process led to higher scavenging radical activity and stronger reducing power in *Ganoderma lucidum* polysaccharides than hot air drying. Together, these studies showed that the methods used for extracted polysaccharide drying affected the properties of polysaccharides, but little is known about how drying raw materials affects the structure and biological activity of the polysaccharides. Drying raw materials may cause the degradation of polysaccharides and the chemical reaction between polysaccharides and other components. In addition, dried litchi pulp is a valuable ingredient in traditional Chinese medicines. We previously showed that polysaccharides from hot air-dried litchi pulp had a lower molecular weight and significantly increased immunostimulatory activity compared with those of fresh pulp [17]. However, whether litchi pulp dried with different methods will result in different effects on its polysaccharides is not clear.

Therefore, the purposes of our present study are to (1) investigate the differences in the physicochemical properties of polysaccharides from litchi pulp dried using VF, VM and HP methods; (2) compare the *in vitro* immunomodulatory activity of the samples to probe the relationship between structure and immunomodulatory activity of the polysaccharides.

## 2. Results and Discussion

### 2.1. Preliminary Characterization of Litchi Polysaccharide-Protein Complexes

#### 2.1.1. The Chemical Compositions of Litchi Polysaccharide-Protein Complexes

The final yields of litchi polysaccharides from vacuum freeze, vacuum microwave and heat pump dried litchi pulp, which were coded as LP-VF, LP-VM and LP-HP, were 3.51%, 3.28% and 3.43%, respectively. The contents of neutral sugar, uronic acid and protein, as well as monosaccharide and amino acids composition were presented in Table 1. Three polysaccharides samples were actually polysaccharide-protein complexes because of the existence of binding protein. LP-VF contained the highest neutral sugar, uronic acid and binding protein content, followed by LP-HP and LP-VM (*p <* 0.05). This is similar to the previous observation that *Ganoderma lucidum* extraction dried with freeze-drying, and had higher polysaccharide content than those dried with other drying methods [18]. These differences were shown to be dependent on the temperature used to dry the litchi pulp, and the drying process itself. Specifically, it may due to the presence of enzymes in the pulp that catalyze various chemical reactions and then alter the composition of constituents. These enzymes are inactivated at −20 °C, while many enzymes, such as hydrolytic enzymes and pectinase, retain activity when litchi pulp is dried at 50–55 °C. Hydrolytic enzymes play a significant role in the degradation of polysaccharides, and pectinase activity has a strong influence on the neutral sugar and uronic acid content [19]. In addition, the microwave heating method causes polarization of polar bonds such as the C–O–C glycosidic linkages, and the reactivity of the molecules is enhanced, which can lead to hydrolysis of polysaccharide chains and breakage of intermolecular hydrogen bonds [20]. These may explain why the LP-VM sample contained the lowest neutral sugar, uronic acid and binding protein content among the three samples from different drying methods employed in this study. However, the influence on the glycosidic linkages, and conformation of polysaccharide-protein complexes, in particular, need further investigation.

Six monosaccharides (rhamnose, arabinose, ribose, mannose, galactose and glucose) were identified in all three litchi polysaccharide-protein complexes (LPCs), but the proportion of each differed significantly between samples (Table 1). The major monosaccharide in LP-VF and LP-VM was glucose, while galactose was more abundant in LP-HP. The percentage contribution of glucose in LP-VF, LP-VM and LP-HP decreased successively while galactose showed the opposite trend (*p* < 0.05). Moreover, there was no significant difference in the contents of mannose and rhamnose among there LPCs (*p* > 0.05). The proportion differences may be related with the drying temperature. When HP drying is carried out at 75 °C, heating effects result in the depolymerization of cell wall polysaccharides and a corresponding increase in pectin-associated sugars such as arabinose and galactose [21].

A total of 16 amino acids were detected within the binding proteins isolated with three LCPs (Table 1). Aspartate, threonine, serine, glutamic, glycine and lysine were significantly more abundant in LP-HP than in LP-VM and LP-VF (*p <* 0.05).

**Table 1:** Chemical compositions of litchi polysaccharide-protein complexes.

Composition	LP-VF	LP-VM	LP-HP
Neutral Sugar (%)	51.51 ± 1.89 ^c^	38.36 ± 0.84 ^a^	49.81 ± 2.32 ^b^
Uronic Acid (%)	6.8 ± 0.26 ^c^	4.23 ± 0.15 ^a^	4.63 ± 0.35 ^b^
Protein (%)	4.2 ± 0.03 ^c^	4.03 ± 0.02 ^a^	4.11 ± 0.04 ^b^
**Monosaccharides Composition (%)**			
Ribose	2.06 ± 0.35 ^a^	2.00 ± 0.62 ^a^	3.43 ± 0.06 ^b^
Rhamnose	0.23 ± 0.04 ^a^	0.23 ± 0.19 ^a^	0.38 ± 0.06 ^a^
Arabinose	9.67 ± 1.56 ^a^	12.79 ± 0.74 ^b^	27.25 ± 1.09 ^c^
Xylose	0.97 ± 0.28 ^a^	0.85 ± 0.19 ^a^	2.04 ± 0.63 ^c^
Mannose	9.69 ± 3.01 ^a^	12.74 ± 2.98 ^a^	13.94 ± 0.21 ^a^
Glucose	59.00 ± 4.32 ^c^	50.75 ± 2.57 ^b^	11.54 ± 0.8 ^a^
Galactose	17.90 ± 0.18 ^a^	21.35 ± 2.76 ^b^	40.08 ± 3.11 ^c^
**Amino Acids Composition (g/100 g)**			
Aspartate	0.243 ± 0.040 ^a^	0.257 ± 0.040 ^a^	0.467 ± 0.095 ^b^
Threonine	0.440 ± 0.030 ^b^	0.290 ± 0.010 ^a^	0.378 ± 0.075 ^b^
Serine	0.337 ± 0.042 ^a^	0.253 ± 0.038 ^a^	0.450 ± 0.131 ^b^
Glutamic	0.367 ± 0.084 ^a^	0.363 ± 0.021 ^a^	0.493 ± 0.040 ^b^
Glycine	0.303 ± 0.032 ^b^	0.227 ± 0.023 ^a^	0.283 ± 0.052 ^b^
Alanine	0.423 ± 0.124 ^a^	0.383 ± 0.462 ^a^	0.333 ± 0.064 ^a^
Valine	0.487 ± 0.080 ^b^	0.313 ± 0.015 ^a^	0.384 ± 0.093 ^a^
Methionine	0.106 ± 0.050 ^a^	0.062 ± 0.036 ^a^	0.100 ± 0.020 ^a^
Isoleucine	0.250 ± 0.045 ^a^	0.200 ± 0.010 ^a^	0.307 ± 0.068 ^a^
Leucine	0.303 ± 0.045 ^b^	0.170 ± 0.044 ^a^	0.273 ± 0.099 ^ab^
Tyrosine	0.087 ± 0.010 ^a^	0.060 ± 0.014 ^a^	0.071 ± 0.018 ^a^
Phenylalanine	0.136 ± 0.037 ^a^	0.097 ± 0.032 ^a^	0.159 ± 0.072 ^a^
Histidine	0.487 ± 0.076 ^a^	0.417 ± 0.021 ^a^	0.460 ± 0.098 ^a^
Lysine	0.393 ± 0.031 ^a^	0.387 ± 0.072 ^a^	0.500 ± 0.017 ^b^
Arginine	0.287 ± 0.087 ^b^	0.075 ± 0.022 ^a^	0.075 ± 0.005 ^a^
Proline	0.237 ± 0.078 ^a^	0.24 ± 0.106 ^a^	0.373 ± 0.071 ^a^

Rows with different letters represent a statistical difference of *p* < 0.05.

#### 2.1.2. Molecular Weight Distribution of Polysaccharide-Protein Complexes

**Figure 1:**
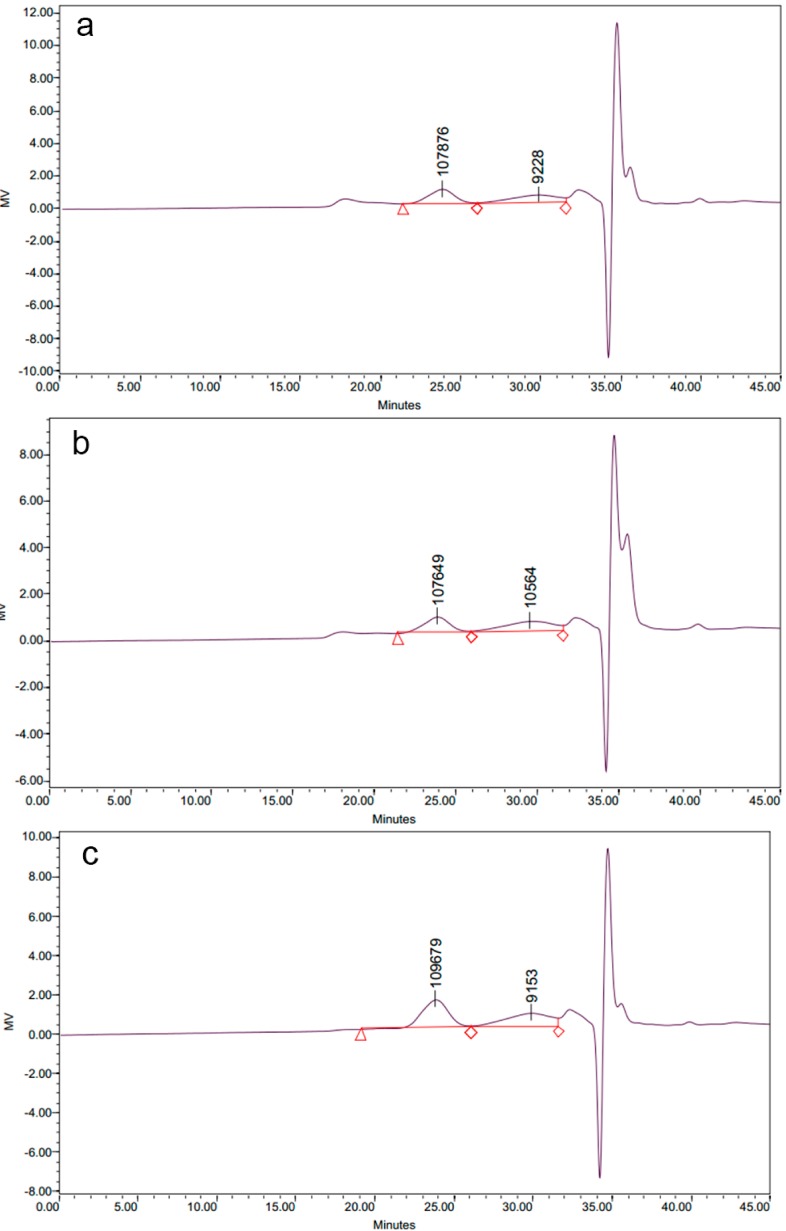
The molecular weight distribution of litchi polysaccharide-protein complexes from vacuum freeze dried pulp (LP-VF) (**a**), litchi polysaccharide-protein complexes from vacuum microwave dried pulp(LP-VM) (**b**), litchi polysaccharide-protein complexes from heat pump dried pulp (LP-HP) (**c**). The average molecular weights of litchi polysaccharide-protein complexes(LPCs) were determined by gel permeation chromatography (GPC).

The molecular weight distribution of all LPCs (Figure 1) consisted of two main peaks for The molecular masses of peak 1 were 107,876 Da, 107,649 Da and 109,679 Da for LP-VF, LP-VM and LP-HP, respectively, while the masses of peak 2 were 9228 Da, 10,564 Da and 9153 Da.

To date, the effects of drying processing on molecular weight distribution of polysaccharides from different materials were variously reported. The pectin polysaccharides from Japanese persimmon fruit degraded into intermediate and then low molecular weight polymer during the sun-drying process [22]. Oven drying, vacuum oven drying, spray drying and freeze drying processes led to decrease of the molecular weight of durian seed gum [23]. Microwave heating induced the degradation of polysaccharide from *Porphyra yezoensis* [24]. However, hot-air and vacuum drying process removed part of the hydration layer of polysaccharides, which disrupted the polysaccharide structure and caused aggregation of polysaccharides from Inonotus obliquus [15]. Our results showed that the three drying methods used in this study—vacuum freeze, vacuum microwave and heat pump dying—resulted in polysaccharides with similar molecular weight distribution in the dried litchi pulp. The discrepancy between our results and the previous research may be due to the difference in materials. We deduce that the drying methods used in this study might not be violent enough to result in degradation or recombination of polysaccharide molecules in litchi pulp.

#### 2.1.3. Spectroscopic Characteristics of Polysaccharide-Protein Complexes

**Figure 2:**
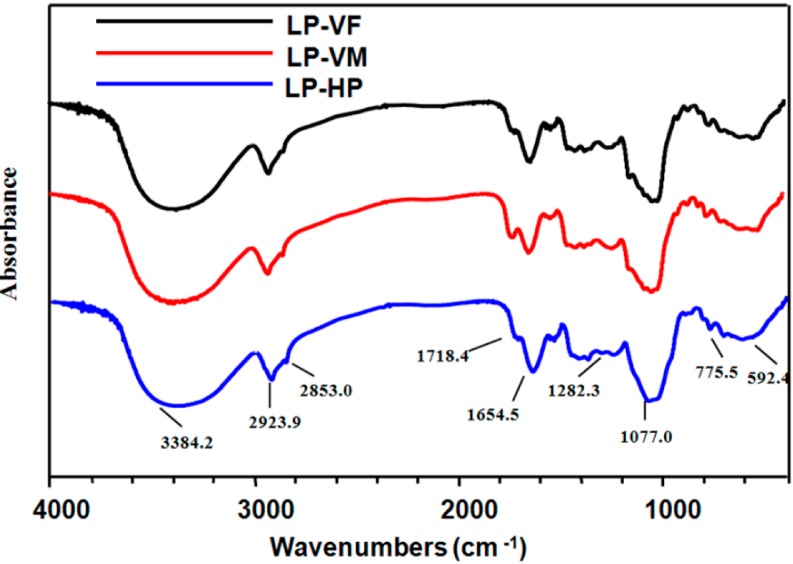
FT-IR spectra of polysaccharide-protein complexes. The FT-IR spectra of litchi polysaccharide-protein complexes were determined using a Fourier-transform infrared spectrophotometer over the frequency range of 4000–400 cm^−1^. LP-VF: Litchi polysaccharide-protein complexes from vacuum freeze dried pulp; LP-VM: Litchi polysaccharide-protein complexes from vacuum microwave dried pulp; LP-HP: Litchi polysaccharide-protein complexes from heat pump dried pulp.

FT-IR experiments showed similar IR absorption profiles for three LPCs (Figure 2), with the characteristic polysaccharide bands including the hydroxyl group bands at 3600–3200 and 1075–1010 cm^−1^, the alkyl group bands around 2923.9 and 2853.0 cm^−1^, and the carboxyl group bands in the 1720–1260 cm^−1^ region (characteristic of uronic acid). The absorption bands between 1100 cm^−1^ and 1000 cm^−1^ were characteristic of C-O-C glycosidic bond vibrations and ring vibrations overlapping with stretching vibrations of the side groups of the C-O-H links. The absorption peaks around 3384.2 and 1654.5 cm^−1 ^ were feature of protein IR peaks [25]. These results confirmed the presence of polysaccharides, proteins and uronic acids in all three LPCs. 

### 2.2. In Vitro Immunostimulatory Activity of Litchi Polysaccharide-Protein Complexes

#### 2.2.1. Effects of Litchi Polysaccharides on Splenocyte Proliferation *in Vitro*

**Figure 3:**
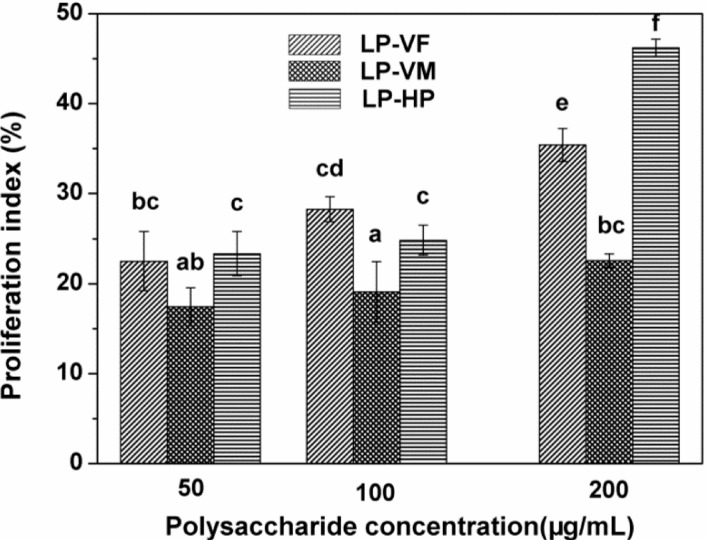
Effects of litchi polysaccharide-protein complexes on the proliferation index of splenocytes at different concentrations (50, 100 and 200 μg/mL). The proliferation indexes were assessed by the MTT assay and expressed as the mean ± standard deviation (*n* = 6). Bars labeled with different letters represent a statistical difference at *p <* 0.05. LP-VF: Litchi polysaccharide-protein complexes from vacuum freeze dried pulp; LP-VM: Litchi polysaccharide-protein complexes from vacuum microwave dried pulp; LP-HP: Litchi polysaccharide-protein complexes from heat pump dried pulp.

Lymphocyte proliferation is a crucial event in the activation cascade of both cellular and humoral immune responses [26]. Therefore, we studied the stimulatory effects of three LPCs on mouse splenocyte proliferation (Figure 3). The proliferation indices were enhanced by 22.49%–35.4%, 17.43%–22.56% and 23.33%–46.21% with LP-VF, LP-VM and LP-HP, respectively. LP-HP exhibited the strongest stimulatory activity, followed by LP-VF and LP-VM at 200 μg/mL (*p <* 0.05). The differences in stimulatory effects of three LPCs may be due to their different chemical structure properties. Lo *et al.* reported that arabinose, mannose, xylose and galactose, but not glucose, of *Lentinula edodes* polysaccharides, played an important role in the activation of macrophage [27]. In addition, protein bound polysaccharides, PSK and PSP, with glutamic and aspartate predominate in the peptide component of these compounds, have been shown to act as potent immunomodulating agents [28]. LP-HP with more galactose, glutamic and aspartate, less glucose may be related with the stronger stimulatory activity than LP-VF and LP-VM.

#### 2.2.2. Effects of Litchi Polysaccharide-Protein Complexes on the Production of NO, IL-6 and TNF-α of RAW264.7 Macrophages

**Figure 4:**
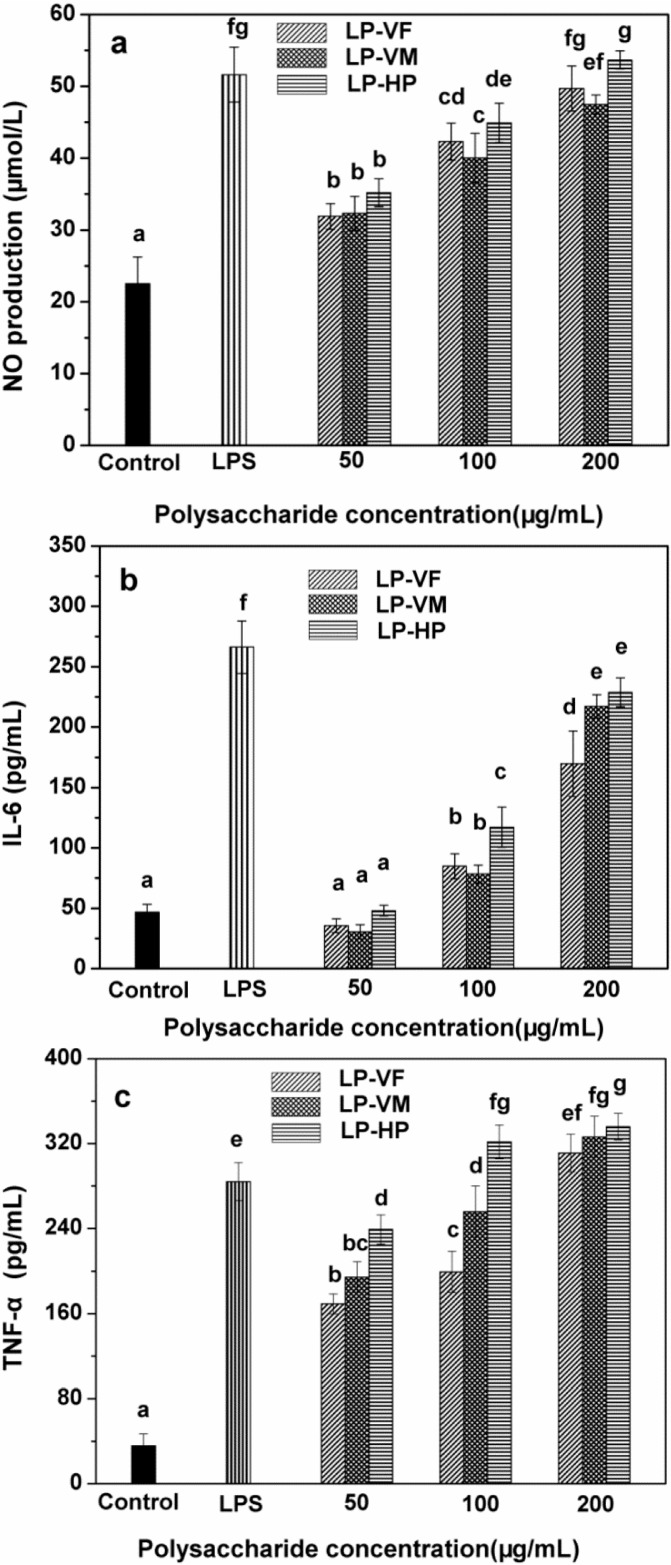
Effects of litchi polysaccharide-protein complexes on the production of NO (**a**), IL-6 (**b**) and TNF-α (**c**) of RAW264.7 macrophages. LPCs (50, 100 and 200 μg/mL) and LPS (5 μg/mL) were added to stimulate RAW264.7 macrophages. NO, IL-6 and TNF-α were measured by ELISA kits. The results were expressed as the mean ± standard deviation (*n* = 6). Bars labeled with different letters represent a statistical difference at *p <* 0.05. LP-VF: Litchi polysaccharide-protein complexes from vacuum freeze dried pulp; LP-VM: Litchi polysaccharide-protein complexes from vacuum microwave dried pulp; LP-HP: Litchi polysaccharide-protein complexes from heat pump dried pulp.

Macrophages are gatekeepers of innate immunity system and exert numerous important functions involving the phagocytosis of microbial pathogens and abnormal cells and the production of cytokines [29]. Cytokines control homeostasis of the organism by regulating cell differentiation, proliferation and apoptosis, as well as defense functions such as immune and inflammatory reactions [30]. Therefore, we studied the effects of LPCs on secretion of NO, IL-6 and TNF-α by mice macrophages (Figure 4). Three LPCs facilitated the production of NO in a dose-dependent manner (Figure 4a). LP-VF, LP-VM and LP-HP stimulated the highest NO production of 49.69, 47.46 and 53.68 μmol/L at 200 μg/mL, respectively, which are comparable with that of 5 μg/mL LPS. LP-HP exhibited stronger stimulatory activity than LP-VF and LP-VM at the same concentrations except at 50 μg/mL (*p <* 0.05).

Moreover, IL-6 secretion was stimulated in a dose-dependent manner by three LPCs (Figure 4b). The highest IL-6 concentrations were 169.74, 217.09 and 228.72 pg/mL when the macrophages were stimulated with LP-VF, LP-VM and LP-HP, respectively. LP-HP showed higher IL-6 stimulatory activity than LP-VF (*p <* 0.05), but was comparable with LP-VM.

Three LPCs augmented TNF-α production significantly (Figure 4c) in a dose-dependent manner at tested concentrations. LP-VF, LP-VM and LP-HP at 200 μg/mL increased the secretion of TNF-α to the highest levels of 311.08, 326.37 and 335.99 pg/mL, respectively, and showed stronger stimulation than 5 μg/mL of LPS (*p <* 0.05). LP-HP showed stronger stimulation than LP-VF and LP-VM at the same concentration (*p <* 0.05).

We previously showed that LPCs from dried litchi pulp exhibited stronger immunomodulatory activity than those from fresh pulp [17]. Therefore, we further investigated the effects of different drying methods on the immunostimulation of LPCs. In this study, LP-HP showed stronger stimulation of spleen lymphocyte proliferation and NO, IL-6, TNF-α secretion of RAW264.7 mice macrophages than LP-VM and LP-VF. The discrepancy of bioactivity could be attributed to the differences in physicochemical properties of three LPCs.

Polysaccharides interact with immune cells via membrane receptors, resulting in stimulation of intracellular signaling cascades that affect immunological responses [31]. The stimulating activities of polysaccharides are correlated with their monosaccharide composition. In this study, LP-HP, mainly containing galactose and arabinose, showed stronger immunoregulatory activity *in vitro* than both LP-VF and LP-VM, in which glucose is the main monosaccharide. It was previously reported that galactose and arabinose played a key role in the immunomodulatory activity of polysaccharides from *Chlorellapyrenoidosa* [32]. Another study on polysaccharides from *Lentinula edodes* reported that arabinose, mannose, xylose and galactose contributed more in the stimulation of macrophages than glucose [27]. In addition to monosaccharides, the presence of binding proteins may also affect the immunomodulatory activity of LPCs. The immunomodulatory and antitumor activity of polysaccharide-protein complexes from *Pleurotus tuber-regium* [33] and from culture filtrates of *Tricholoma lobayense* [34] were correlated with the amino acid composition, specifically with aspartate, glutamic, serine, glycine and threonine content. Our results are consistent with the previous studies. LP-HP, which contained more aspartate and threonine, showed stronger immunoregulatory activity than LP-VF and LP-VM. Increased expression of IL-6 and TNF-α is considered to be an important signal of higher immunoactivity. Both IL-6 and TNF-α were demonstrated to be a growth factor of activated B cell to induce the final maturation to antibody-forming plasma cells. IL-6 acts on mitogen-activated B cells to induce IgM, IgG and IgA production [35,36]. Enhancing immnoactivity is very important for immunocompromised people, such as cancer patients on radio- or chemotherapy. Therefore, many polysaccharides have been reported to show immunomodulatory properties by promoting IL-6 and TNF-α production [37,38].

#### 2.2.3. Effects of Polymyxin B (PB) on the Biological Activity of Litchi Polysaccharide-Protein Complexes

**Figure 5:**
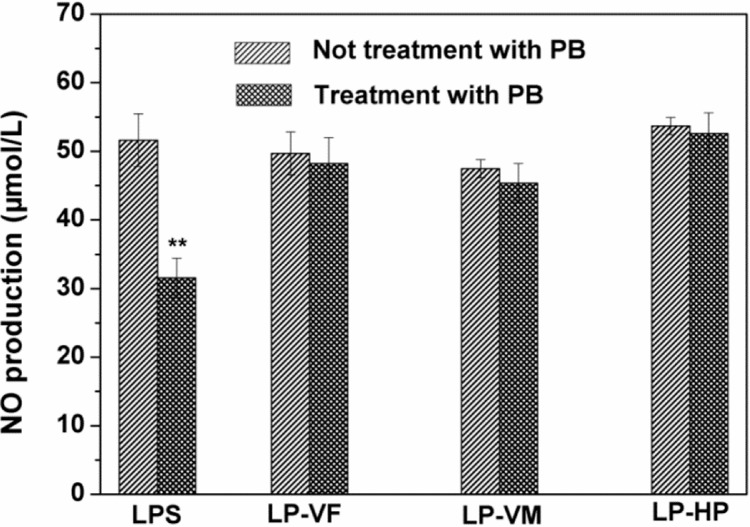
Effects of polymyxin B on the biological activity of litchi polysaccharide-protein complexes. LPCs (200 μg/mL) and LPS (5 μg/mL) with or without PB (10 μg/mL) were added to stimulate RAW264.7 macrophages. NO were measured by ELISA kits. The results were expressed as the mean ± standard deviation (*n* = 6). ** *p <* 0.01 compared with LPS treatment without PB. LP-VF: Litchi polysaccharide-protein complexes from vacuum freeze dried pulp; LP-VM: Litchi polysaccharide-protein complexes from vacuum microwave dried pulp; LP-HP: Litchi polysaccharide-protein complexes from heat pump dried pulp.

To ensure that the immunostimulatory effects of three LPCs were not due to endotoxin contamination, RAW264.7 mice macrophages were co-stimulated with Polymyxin B together with LPCs or LPS (Figure 5). PB markedly reduced NO production by macrophages stimulated with LPS (*p <* 0.01), but had no effect on the activity of LPCs, which confirmed that the above mentioned bioactivity of LPCs were exerted by LPCs themselves but not endotoxin contamination.

## 3. Experimental Section

### 3.1. Materials and Chemicals

#### 3.1.1. Chemicals and Reagents

Standard dextrans, rhamnose (Rha), arabinose (Ara), glucose (Glc), xylose (Xyl), galactose (Gal), mannose (Man), penicillin-streptomycin solution, lipopolysaccharide (LPS), polymyxin B and 3-(4,5-dimethylthiazol-2-yl)-2,5-diphenyltetrazolium bromide (MTT) were purchased from Sigma Chemical Co. (St. Louis, MO, USA). RPMI-1640 medium, new bovine calf serum and Hank’s balanced salt solution (HBSS) were purchased from Gibco Life Technologies (Grand Island, NY, USA). Enzyme-linked immunosorbent assay (ELISA) kits for IL-6 and TNF-α were purchased from R & D Systems (Minneapolis, MN, USA). All other reagents were of analytical grade.

#### 3.1.2. Cells and Animals

RAW264.7 macrophage cell line was purchased from the Experimental Animal Laboratory of Sun Yat-Sen University (Guangzhou, China). Cells were cultured in RPMI-1640 medium containing 10% fetal calf serum, 100 U/mL penicillin and 100 μg/mL streptomycin at 37 °C and 5% CO_2_.

BALB/c mice (male, 20.0 ± 2.0 g) of 6–8 weeks old were provided by the Experimental Animal Laboratory of Sun Yat-Sen University. The mice were acclimatized for 1 week before use in this study. All animal treatments were performed in accordance with the Guide for the Care and Use of Laboratory Animals. All animal protocols were approved by the institutional animal care and use committee of Sun Yat-Sen University (Guangzhou, China).

### 3.2. Drying Process

Fresh Litchi (Huai-zhi cultivar) fruits were provided by the Pomology Research Institute of Guangdong Academy of Agricultural Sciences (Guangzhou, China). Ripe Litchi fruits of uniform size and free from visual defects were selected. Fresh fruits were peeled; 1 kg of fresh pulp was spread evenly on petri dishes, and dried to a moisture content of 22% ± 1% using either VF drying, VM drying or HP drying. For VF drying, pulp was lyophilized in a vacuum flask at 0.125 mbar and −20 °C using a freeze-dryer (Eyela, Tokyo, Japan). For VM drying, pulp was dried in a microwave vacuum-dryer (Kailing, Guangzhou, China) at 2 kW and −0.09 MPa using an intermittent drying process in which the temperature was maintained between 50 and 55 °C. For HP drying, a heat pump-dryer (Kailing, Guangzhou, China) at 75 °C was used at a flow velocity of 1 m/s. Dried pulps were packed and stored at −20 °C until needed.

### 3.3. Preparation of Polysaccharide-Protein Complexes

Dried pulp samples were minced into small pieces and soaked in 80% ethanol (1:4, w/v) at 4 °C for 24 h to remove pigments, monosaccharides and oligosaccharides. After filtration through a Whatman No. 1 paper, residues were homogenized and extracted three times with distilled water (1:20, w/v) at 50 °C for 6 h. The aqueous extracts were filtered and concentrated to one fifth of the initial volume in a vacuum evaporator (Eyela, Tokyo, Japan) at 50 °C. Proteins were removed using Sevag reagent [19], and polysaccharides were precipitated with absolute ethanol (1:4, v/v) for 24 h at 4 °C. Precipitates were collected by centrifugation at 3000× *g* for 20 min, and washed successively with acetone and petroleum ether. The precipitated polysaccharides were then lyophilized. Polysaccharides obtained from VF dried, VM dried and HP dried litchi pulp were named as LP-VF, LP-VM and LP-HP, respectively. Three LPs were stored in a desiccator at room temperature until needed.

### 3.4. Preliminary Characterization of Litchi Polysaccharide-Protein Complexes

#### 3.4.1. Analysis of the Chemical Characteristics

The neutral polysaccharide content was determined using the phenol-sulfuric acid method [39] and expressed as glucose equivalents. Protein concentration was determined using the Bradford assay with a bovine serum albumin (BSA) standard curve [40], and the uronic acid content was determined using the modified m-hydroxydiphenyl method with galacturonic acid standards [41].

GC-MS was used for identification and quantification of monosaccharides [23]. Briefly, 40 mg of polysaccharides were hydrolyzed in sealed glass tubes using 2 M H_2_SO_4_ (10 mL) at 100 °C for 6 h. After neutralizing the residual acid with BaCO_3_, the hydrolysates were filtered through 0.2 μm syringe filters (Whatman, Sanford, UK), dried under a stream of N_2_ and mixed with hydroxylamine hydrochloride (70 mg) and pyridine (5 mL) at 90 °C for 60 min. Next, 5 mL acetic anhydride was added and acetylation proceeded at 90 °C for 30 min. The acetylated hydrolysates were extracted with trichloromethane and evaporated under a stream of N_2_. The final product was analyzed using a 6890 GC-MS instrument (Agilent Technologies Co., Ltd., Colorado Springs, CO, USA) equipped with a DB-1 column and an Agilent 5973 MS detector. The following temperature programme was adopted. The temperature of column was set at 100 °C initially, and then increased to 280 °C at a rate of 10 °C/min and kept at 280 °C for 15 min. The injection temperature was 280 °C and the temperature of mass spectrometer ion source was 230 °C.

The amino acid composition of LPCs was analyzed as described [42]. Amino acids were released from the complexes by hydrolysis using 6 M HCl at 110 °C for 22 h in a vacuum-sealed tube, and liberated amino acids were determined using an 835-50G automatic amino acid analyzer (Hitachi L-8900, Tokyo, Japan).

#### 3.4.2. Determination of Molecular Weights

The average molecular weights of LPCs were determined by gel permeation chromatography (GPC), which was performed on a Sephacryl S-300HR column (1.6 × 70 cm) with detection limit of 24 μg. Standard dextran including T-4 (molecular mass, 4 × 10^3^ Da), T-10 (1 × 10^4^ Da), T-40 (4 × 10^4^ Da), T-70 (7 × 10^4^ Da), T-500 (5 × 10^5^ Da), and T-2000 (2 × 10^6^ Da) were used as molecular mass markers.

#### 3.4.3. Analysis of FT-IR Spectroscopy

FT-IR spectra were recorded on a Nexus 5DXC FT-IR (Thermo Nicolet, Austin, TX, USA) in the frequency range 4000−400 cm^−1^. The samples were mixed with potassium bromide (KBr) powder and pressed into a 1 mm thick pellet for FT-IR measurement.

### 3.5. In Vitro Immunostimulatory Activity Assay of Litchi Polysaccharides-Protein Complexes

#### 3.5.1. Determination of Mouse Splenocyte Proliferation

Spleens were removed from sacrificed BALB/c mice and minced in sterile phosphate buffered saline (PBS). Splenic cells were harvested using a sterilized stainless steel mesh (200 meshes) at room temperature. Red blood cells were lysed with hemolytic Gey’s solution, and the remaining cells were washed twice and resuspended in RPMI 1640 complete medium containing 10% fetal calf serum, 100 U/mL penicillin and 100 μg/mL streptomycin. The trypan-blue dye exclusion test showed that more than 95% of cells were viable.

The splenocyte proliferation assay was performed as previously described [43]. In the mitogenic test, 5 × 10^5^ cells per well in 0.1 mL medium were seeded into a 96-well plate. Cells were stimulated with LPs at a final concentration of 0, 50, 100 and 200 μg/mL for 68 h. Next, 20 μL of MTT (5 mg/mL) was added to each well and cells were cultured for 4 h. Acidified isopropyl alcohol (100 μL) was added and incubated for 12 h at 37 °C to dissolve the formazan crystals. The absorbance at 570 nm was recorded using a microplate reader (Tecan Infinite Pro 200, Männedorf, Switzerland). The stimulation index (%) of splenocyte proliferation was calculated using the following formula:

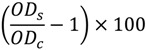
(1)
where, ODs, represent the absorbance of the sample group; ODc, represent the absorbance of control group.

#### 3.5.2. Determination of NO and Cytokine Production of Macrophage

RAW264.7 macrophages (5 × 10^5^ cells/well) suspended in complete RPMI-1640 medium were plated into 24-well culture plates and incubated for 3 h. After washing out the non-adherent cells, 500 μL/well of stimulant (the final concentration of polysaccharides-protein complexes: 0, 50, 100 or 200 μg/mL, that of LPS was 5 μg/mL) was added in and incubated for another 24 h. Next, 150 μL of culture medium was transferred from each well into a 1.5 mL centrifuge tube containing 10 μL ZnSO_4_ solutions (300 mg/mL). The mixture was centrifuged at 3000× *g* for 10 min and the supernatant was used for NO quantitation. Nitrite accumulation in the culture medium was used as an indicator of NO production based on the Griess reaction. A total of 100 μL of supernatant was incubated with an equal volume of Griess solution (1% sulfanilamide in 2.5% phosphoric acid, 0.1% N-(1-naphthyl)- ethylenediamine in 2.5% phosphoric acid) at 25 °C for 10 min. The absorbance at 540 nm was measured and the concentration of NO^2−^ was determined using least-squares linear regression analysis and a sodium nitrite standard curve [44].

RAW264.7 macrophages in the exponential growth phase (5 × 10^5^ cells/well) suspended in complete RPMI-1640 medium were placed into 24-well culture plates and incubated for 6 h. After washing off non-adhered cells, 500 μL of LPs (0, 50, 100 or 200 μg/mL final concentration) or 5 μg/mL LPS were added and incubated for a further 48 h. The culture media was centrifuged at 3000× *g* for 10 min at 4 °C, and IL-6 and TNF-α in the supernatant was quantified using the double-antibody sandwich ELISA according to the manufacturer's instructions. Three replicates were performed for each treatment.

#### 3.5.3. Determination of Endotoxin Contamination of Litchi Polysaccharides-Protein Complexes

Potential endotoxin contamination of LPCs was investigated by testing the NO emission from RAW264.7 mice macrophages as described previously. Macrophages were placed in a 24-well plate and incubated for 3 h. After washing off non-adhered cells, 500 μL of stimulant (200 μg/mL LPCs, 5 μg/mL LPS, a mixture of 200 μg/mL LPs and 10 μg/mL polymyxin B, or a mixture of 5 μg/mL LPS and 10 μg/mL PB) dissolved in complete RPMI-1640 medium was added to each well and cells were incubated for a further 24 h. Next, 150 μL of culture medium was centrifuged at 3000× *g* for 10 min with ZnSO_4_ to obtained the supernatant was used for NO quantitation with Griess reaction. The absorbance at 540 nm was measured and the concentration of NO^2−^ was determined using least-squares linear regression analysis and a sodium nitrite standard curve.

### 3.6. Statistical Analysis

Data were expressed as means ± standard deviation (SD). The significance of the difference was evaluated by one-way ANOVA followed by the Student-Newman-Keuls test using SPSS 19.0 software, and a *p*-value of 0.05 was chosen as the threshold for significance.

## 4. Conclusions

LPCs prepared by VF drying, VM drying and HP drying methods showed differences in their physicochemical properties and immunomodulatory activities. LP-HP contained major galactose and more aspartate and glutamic in binding protein. LP-HP also showed relatively higher stimulation of spleen lymphocyte proliferation and NO, IL-6, TNF-α secretion of RAW264.7 mice macrophages than LP-VM and LP-VF. Therefore, heat pump-drying appears to be the best drying method for preparation of LPCs with the strongest immunomodulatory properties. 
